# Associations between *ACTN3* and OPPERA pain-related genes in malocclusion

**DOI:** 10.1186/1744-8069-10-S1-P4

**Published:** 2014-12-15

**Authors:** JH Godel, BF Foley, R Nicot, MJ Horton, ER Barton, J Ferri, G Raoul, AR Vieira, JJ Sciote

**Affiliations:** 1 Department of Orthodontics Temple University, Philadelphia, PA 19122, USA; 2 Department of Oral & Maxillofacial Surgery, Université of Lille Nord de France, Lille, France; 3 Department of Anatomy & Cell Biology, University of Pennsylvania, Philadelphia, PA 19122, USA; 4 Department of Oral Biology, University of Pittsburgh, Pittsburgh, PA 19122, USA

## Background

We have investigated an orthognathic surgery population to determine how variation in masticatory muscle gene expression and genotype plays a key role in development of both jaw-deformation malocclusion and temporomandibular joint disorders (TMD). A gene of particular interest is *ACTN3* since the common R577X polymorphism results in α-actinin-3 protein loss, reduced myofiber Z-disc structural integrity in skeletal muscle and decreased osteoblast/osteoclast activity in bone formation. Secondly, since the prevalence of TMD in this population is quite high (30%) we sought to determine if genes related to pain processes─previously identified in the Orofacial Pain: Prospective Evaluation and Risk Assessment Study (OPPERA) were differentially expressed.

## Methods

After obtaining masseter muscle and saliva-DNA samples from subjects during orthognathic surgery, we identified associations between genotype, muscle gene expression, fiber type properties, malocclusion classification, facial asymmetry, gender and TMD. Genotype and gene message quantities were determined using TaqMan chemistry. Morphometry of muscle fiber types was conducted on tissue cross sections stained with myosin heavy chain–specific antibodies using NIH Image software. Jaw Pain and Function questionnaire and clinical examinations were used to diagnose TMD. Malocclusion diagnosis was determined by the type of treatment plans executed during surgery.

In a separate pilot analysis muscle samples were analyzed for gene expression differences on Affymetrix HT2.0 microarray expression chips containing 70, 534 transcripts. Principal Components Analysis and False Discovery Rate corrections were applied to comparisons with Partek Genomics Suite software.

## Results

We identified associations between *ACTN3* genotypes and skeletal class II (p=0.003) and deep bite (p=0.03) malocclusions, masseter muscle fiber type properties (p=0.02) and an almost significant association for presence of TMD, which was often limited to masticatory muscle pain (p=0.08). Global gene expression analysis identified significant differences for approximately 200 OPPERA pain process genes in subjects with asymmetry and TMD, compared to subjects with malocclusion only. Differential expression in masseter muscle for one of these genes, *CACNA2D1* (voltage-dependent calcium channel subunit alpha-2/delta-1, active in neuropathic pain), was confirmed with additional quantitative RT-PCR experiments by gender (p=0.0008) and between women with and without myalgia (p=0.05).

## Conclusions

These results indicate that *ACTN3* genotypes make significant contributions in the development of malocclusion as a musculoskeletal condition. Dentofacial deformity subjects, especially females, have a high prevalence for TMD, diagnosed clinically as masticatory muscle myalgia. Differences in α-actinin-3 protein levels may predispose muscle to contraction-induced damage, or altered calcineurin-mediated nociception.

